# Non-invasive multimodal optical coherence and photoacoustic tomography for human skin imaging

**DOI:** 10.1038/s41598-017-18331-9

**Published:** 2017-12-21

**Authors:** Zhe Chen, Elisabet Rank, Kristen M. Meiburger, Christoph Sinz, Andreas Hodul, Edward Zhang, Erich Hoover, Micheal Minneman, Jason Ensher, Paul C. Beard, Harald Kittler, Rainer A. Leitgeb, Wolfgang Drexler, Mengyang Liu

**Affiliations:** 1 Center for Medical Physics and Biomedical Engineering, Medical University of Vienna, Währinger Gürtel 18-20, AKH 4L, 1090 Vienna, Austria; 2 Dipartimento di Elettronica e Telecomunicazioni, Biolab, Politecnico di Torino, Corso Duca degli Abruzzi 24, 10129 Torino, Italy; 3 Department of Dermatology, Medical University of Vienna, Währinger Gürtel 18-20, AKH 7J, 1090 Vienna, Austria; 4 Department of Medical Physics and Biomedical Engineering, University College London, Gower Street, WC1E 6BT, London, UK; 5 Insight Photonic Solutions, Inc., 2650 Crescent Drive, Number 201, Lafayette, CO 80026 USA

## Abstract

The cutaneous vasculature is involved in many diseases. Current clinical examination techniques, however, cannot resolve the human vasculature with all plexus in a non-invasive manner. By combining an optical coherence tomography system with angiography extension and an all optical photoacoustic tomography system, we can resolve in 3D the blood vessels in human skin for all plexus non-invasively. With a customized imaging unit that permits access to various parts of patients’ bodies, we applied our multimodality imaging system to investigate several different types of skin conditions. Quantitative vascular analysis is given for each of the dermatological conditions to show the potential diagnostic value of our system in non-invasive examination of diseases and physiological processes. Improved performance of our system over its previous generation is also demonstrated with an updated characterization.

## Introduction

Skin, as the largest organ in human, uses about 5% of the total blood flow to support all its physiological functions^1^. The cutaneous vasculature is not only the main target of pathologic processes in vasculitis^2^ and vasculopathies, but is also involved in other common inflammatory skin diseases such as psoriasis and eczema. In plaque psoriasis, the capillaries in the papillary dermis are widened, elongated and tortuous^3^. Several different types of hand eczemas exhibit different vascular patterns^4^. In addition to inflammatory diseases, neoplastic skin diseases^5^ and endocrine diseases like diabetes mellitus^6^ are all related to skin vessels’ morphology. Several types of skin cancer, such as melanoma^7^ and basal cell carcinoma^8^ are typified by specific cancer cell – vessel interactions. The visualization of the cutaneous vasculature is hence of great interest not only to clinical dermatology, but also to basic medical sciences and cancer research. Non-invasive acquisition of the 3D morphology of cutaneous blood vessels will also enable monitoring of treatment effects in various skin diseases^9^.

Many methods have been proposed for the visualization of cutaneous vessels. Dermatoscopy, for example, can evaluate blood vessel architecture and morphology, which has been used to diagnose inflammatory and neoplastic skin diseases^10^. However, it mainly serves as a tool for pigmented skin lesion inspection and not for skin vasculature imaging^11^. Skin biopsies can be visualized by methods such as high-resolution episcopic microscopy to show the complete blood vessel network in 3D^12,13^. However, its disadvantages are that it is invasive, and the reconstruction process is time consuming. Alternative approaches such as magnetic resonance imaging can visualize major human skin blood vessels^14^, but they do not have the fine resolution to reveal the complete cutaneous vasculature from capillary loops to deep dermal plexus. X-ray, computed tomographic angiography and Doppler ultrasonography have been explored to visualize human skin blood vessels, but all require intravascular contrast agents or injection of inert gas^5,15,16^.

To achieve non-invasive skin vasculature imaging using endogenous contrast, biomedical optical imaging could be an option. Scattering and absorption are the two categories of light – tissue interaction^17^ and optical imaging modalities based on both of these have been explored in angiography or vasculature imaging in humans. Using scattering contrast, optical coherence tomography angiography (OCTA) can detect blood vessels by sifting out the vessel pixels corresponding to moving red blood cells in a tomogram from the relatively static tissue^18^. Application of OCTA is emerging apace. After successful translation into ophthalmology, in skin, OCTA has also been employed in wound healing assessment^19^, scar vasculature assessment^20^, optical clearing evaluation^21^, multi-contrast imaging^22^ and detection of vascular changes in nevus and melanoma^23^, etc. The use of back scattering light for interference, however, limits OCTA’s penetration depth to ~1–2 mm^24^. Photoacoustic tomography (PAT) operates in the scattering regime, but its resolution is not limited by scattering and is capable to map blood vessels that are situated deeper in biological tissues^25^. Using fiber bundle illumination and a spherically focused transducer, PAT was demonstrated to show the dermal vessels in a healthy volunteer^26^. To visualize individual capillaries, however, PAT has technical challenges. Firstly, a broadband acoustic detector is necessary because the capillaries emit photoacoustic pulses with frequency above 100 MHz while lower frequency components below 25 MHz are also needed for deeper vessels^27^; secondly, even with an ultrawideband system spanning from 20 MHz to 200 MHz, resolving small vessels is only partially successful^28^ due to the limited resolution and sensitivity of PAT; thirdly, since both melanin and hemoglobin contribute to photoacoustic signals, for vessels located in close proximity to skin pigments, differentiation between these two endogenous absorbers could be challenging^15^. The third issue could be particularly problematic for resolving the superficial plexus in darker skin complexions.

Being aware of the limits of OCTA and PAT, combining these two modalities gives access to complementary information. However, the opaque nature of most piezoelectric transducers and their bulky sizes make implementing a PAT system into optical coherence tomography (OCT) very challenging^29^. All optical detection PAT, which works in the backward mode, circumvents the configuration problem and has been demonstrated to work using the same probe with OCT. In the beginning, most efforts were directed towards combining OCT with PAT to visualize the epidermis, dermis and blood vessels^30^. Although the complementarity is confirmed in dermatological imaging using a dual modality OCT/PAT scanner^4^, vessels with diameters less than several tens of micrometers are not visible due to the sensitivity and resolution of the PAT system used. After a phase stable swept source was successfully employed in an OCTA system for human skin imaging^31^, we demonstrated in a previous work that the same region imaged by OCTA and PAT can be co-registered using a transition zone in which both modalities visualize the same blood vessels in human skin^9^. We then merged OCT, OCTA and PAT into one system and mounted the scanner to an articulated arm to access various parts of the body^15^. In this paper, we introduce for the first time an upgraded OCT/OCTA/PAT system, whose components are all assembled into a mobile cart. A rack with all degrees of freedom for the scanner probe and a movable cart allow the system to be easily transferred between labs and clinics for outpatient imaging. We applied the system to image several different types of skin diseases including nevus araneus, basal cell carcinoma and a scar after surgery. Based on previously reported vessel quantification methods for OCTA^32–34^ and PAT^35^, we provide quantitative analysis of the vasculature for these conditions, including vascular morphological and tortuosity parameters such as the vascular density (VD), inflection count metric (ICM) and sum of angles metric (SOAM), etc., using a skeletonization blood vessel quantification algorithm^35^.

## Materials and Methods

We use a multimodal OCT/OCTA/PAT system in the experiments. The basic principles and algorithms used for OCT and OCTA were described in detail in a previous work^31^. In brief, we use a phase stable swept source (SLE-101, Insight Photonic Solutions, CO, US) to perform an OCTA scan with a gate number of 4, meaning that the same frame is scanned consecutively by 4 times. The extraction of vasculature using OCTA is given by Equation (1),

1 $$A(x,y,z)={\frac{1}{3}\sum _{i=0}^{3}|{\mathrm{log}}_{10}(T{(x,z)}_{i+1})-{\mathrm{log}}_{10}(T{(x,z)}_{i})||}_{y}$$

where x and y are the fast and slow scanning axes, respectively; z is the depth axis; A represents the angiographic tomogram; T is the OCT tomogram at position y. Here we use the intensity based algorithm for its fast processing speed. The deeper vessels that are attainable via phase based algorithm^31^ are unnecessary for the multimodal system in that the deeper vessels are better visualized using PAT. Previously, we used a preamplifier in a chassis for the OCTA system because of the fixed dynamic range of the data acquisition device (DAQ) (ATS9360, AlazarTech, Pointe-Claire, Canada). In our upgraded system, to reduce the size of the cart and to simplify assembling, we directly use the built-in preamplifier in the dual balanced detector (DBD) (096214, Insight Photonic Solutions, CO, US). To confirm whether this practice affects the performance of the OCT system, we measured the sensitivity by placing a mirror in the sample arm of the OCT system and placing a neutral density filter in front of the mirror to avoid signal saturation. The calculation of sensitivity follows Equation (2),

2 $$Sensitivity=10\,{\mathrm{log}}_{10}{(\frac{{V}_{peak}}{\sigma })}^{2}+2{ND|}_{dB}$$

where V_peak_ is the peak amplitude from the reconstructed A line with the mirror being the sample; σ is the standard deviation of a reconstructed A line without the mirror; ND is the attenuation in dB by the neutral density filter. We find that the sensitivity is still 103 dB, comparable to that of the previously published system^15^. Similarly, with a mirror in the sample arm, we reconstruct the A line of the mirror and assuming the coating of the mirror to be one order of magnitude smaller than the axial resolution, we fit the A line to a Gaussian function and use the full width at half maximum as the axial resolution, which is 27 µm in air. The lateral resolution of the OCT system is measured by imaging a USAF resolution target with chrome coating. Using the reconstructed *en face* view, we choose an edge from the target and calculate the edge spread function. Then the line spread function is derived from the derivative of the edge spread function. After fitting the line spread function to a Gaussian curve, we use the full width at half maximum of the fitted Gaussian function as the lateral resolution in OCT, which is 55 µm.

In the PAT sub-system, an all optical detection method is employed^36^. Instead of using piezoelectric acoustic sensing, the optical detection method uses a Fabry-Perot interferometer for photoacoustic pulse detection. In our application, it involves an excitation laser and an interrogation laser (Tunics T100S-HP-CL, Yenista Optics, Lannion, France). For some of the results shown in this paper before the cart-based system was assembled, a benchtop excitation laser (VersaScan/BB/HE, GWU-Lasertechnik Vertriebsges.mbH, Erftstadt, Germany) was used. But due to the requirement of a bulky power supply unit for its pumping source (Quanta-Ray Pro-270–50, Spectra-Physics, CA, US), a sizable external chiller (OWT 11, KKT Kraus, Kasendorf, Germany) for pressure exchange, two additional air conditioners and nitrogen bottles, it is not possible to use this excitation source in a cart-based system. Upgrade of the system was accomplished using a relatively compact excitation source (SpitLight 600 OPO, INNOLAS, Krailling, Germany). The new source emits pulses from 680 nm to above 1 µm with a pulse width of 6 ns. The output aperture comes with a condenser for easier coupling into multimode fibers. To further reduce the size of the cart, the DBD for OCT and the photodetector with built-in amplifier and filter are powered by a single power supply unit (TTi EL302RT, Aim-TTi, Cambridgeshire, UK). For easier connection, we also use a new connector block (NI BNC-2110, Nation Instruments, TX, US) to replace the previous one for the PAT system. Another feature of the cart-based system is that the OCT/OCTA and PAT modalities are operated in one single workstation, significantly lessening complexity for using the combined system for clinicians.

**Figure 1:**
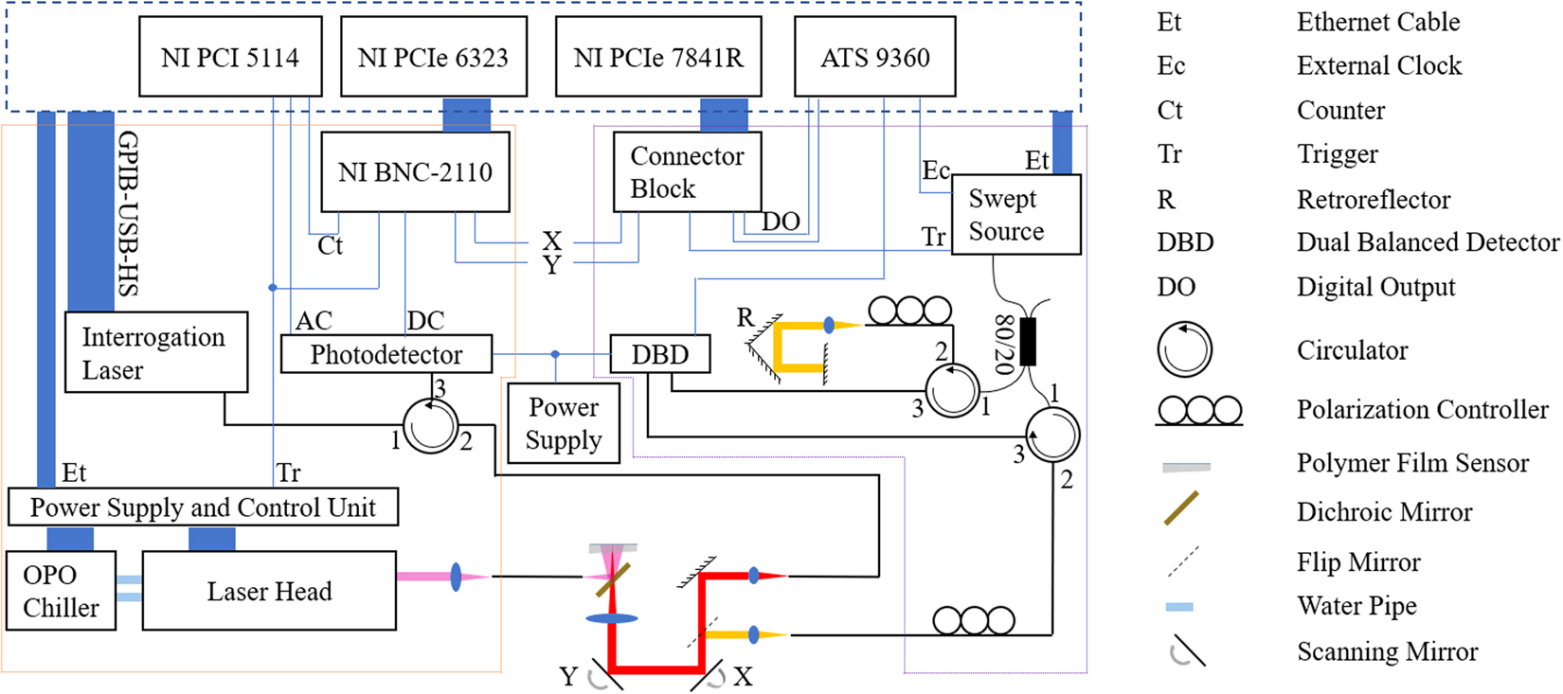
Schematic of the cart-based OCT/OCTA/PAT system. Blue, orange and purple dashed lines encircle the workstation with all the DAQ cards, the PAT and the OCT components, respectively.

Figure 1 shows a schematic of the cart-based OCT/OCTA/PAT system. The blue dashed line encircles the single workstation for the whole system. The OCT swept source communicates with the workstation through an Ethernet cable. The same source supplies the sample clock to the DAQ (ATS9360) and the A line trigger signal to a field programmable gate array (NI PCIe 7841 R, National Instruments, TX, US) through a customized connector block. Synchronization between data acquisition and scanning mirror (6210 H, Cambridge Technology, MA, US) movement is achieved by digital lines between the field programmable gate array and the DAQ. The output from the swept source is centered at 1340 nm with 37 nm bandwidth. It goes through an 80/20 fiber coupler with 80 percent of the power going to the sample arm (about 6.2 mW incident upon sample). The backscattered light from the sample arm interferes with the back-reflected light from the reference arm in the DBD and is acquired by the DAQ. For the PAT part, the excitation laser also connects with the workstation through an Ethernet cable while the interrogation laser is controlled using a high speed GPIB-USB cable. The scanning mirrors scan the polymer film sensor over a 1 cm × 1 cm range with 70 µm increment in both x and y directions. During pretuning, the intensity from the back-reflected beam is then detected by a customized photodetector and the converted voltage signal is sent through the DC output of the photodetector to a DAQ (NI PCIe 6323, National Instruments, TX, US). The optimum bias wavelength, which is the interrogation wavelength corresponding to the highest sensitivity on each scanning point, is then calculated from the interferometer transfer function. The output from the excitation laser is firstly coupled into a multimode fiber. The other end of the fiber is clamped in the probe. The output of the multimode fiber is directed at 45° towards a customized dichroic mirror which reflects the excitation beam (680 nm) and transmits the interrogation beam (telecom range). The excitation beam’s fluence on the sample is about 4 mJ/cm^2^ at a repetition rate of 50 Hz. For each excitation pulse, one point on the sensor is interrogated. The photoacoustic pulse modulated reflected interrogation light intensity is detected by the photodetector, with the amplified voltage signal detected by a DAQ (NI PCI 5114, National Instruments, TX, US). Data acquisition in PAT is synchronized with mirror scanning through a DAQ with analog generation function (NI PCI 6323), which are both triggered per pulse by the excitation laser.

**Figure 2:**
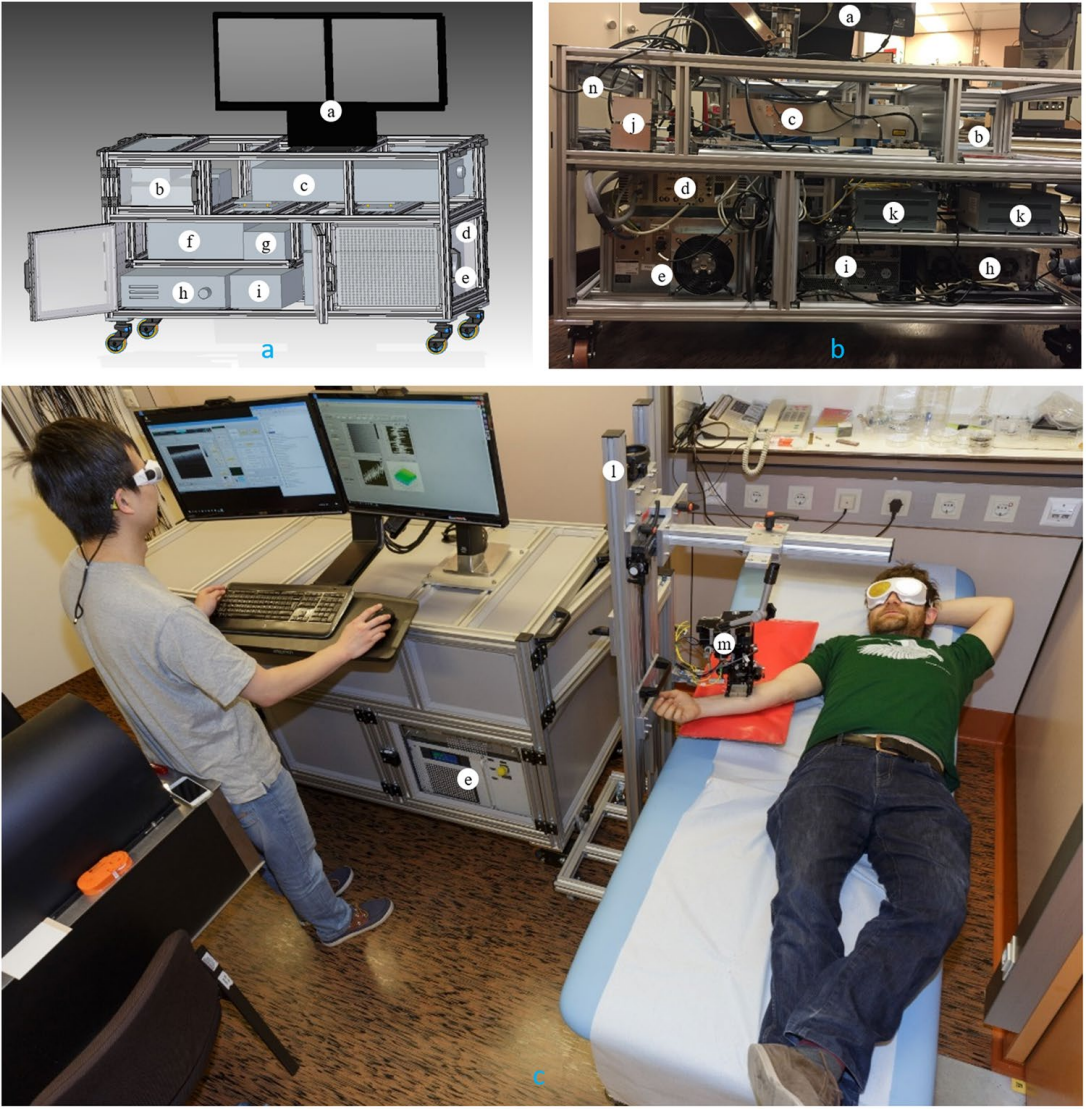
(**a**) Design of the cart. (**b**) Photo of the back side of the cart during assembling. (**c**) Illustration of the system during imaging. (**a**) Dual monitors; (**b**) storage compartment; (**c**) excitation laser head; (**d**) power supply and control unit of the excitation laser; (**e**) chiller of the excitation laser; (**f**) breadboard for OCT optics; (**g**) swept source; (**h**) PAT interrogation laser; (**i**) workstation; (**j**) servo boards for scanning mirrors; (**k**) power supply for servo boards; (**l**) rollable rack with an articulated arm; m: OCT/OCTA/PAT probe; n: compartment for probe storage.

For both OCT/OCTA and PAT, a scanning range of 1 cm × 1 cm is used for all experiments. In OCT/OCTA the pixel size in x and y directions is 512 with a total acquisition time of about 10 s. PAT scan is relatively slow, for which we use 70 µm step size in both x and y directions, yielding a data acquisition time of about 8 minutes. The upgraded system employed in the experiment follows the European Commission Medical Device Directive. Figure 2(a) shows the design of the cart. The frame is made of aluminum. Each compartment is covered by plastic panels. Some panels are either perforated or transparent for ventilation and monitoring purposes. The whole cart is mounted on four weight bearing casters. The excitation laser head (item c in the figure) and its related fiber launching system are bolted to two separate spring-loaded metal plates for vibration damping during transportation and operation. The cart only requires a mains cable connection to power all the devices and an Ethernet cable for data transfer. The size of the cart, excluding the dual monitors and their mount, is 73 cm × 178 cm × 102 cm (width × length × height, not considering the handles for the cart). During transportation, the probe (item m in the figure) is stored inside the compartment in front of the laser head (item n in the figure). Before patient measurement, the probe (m) is taken out of the compartment (n) and is mounted to the mobile rack with an articulated arm (item l in the figure). An illustrative figure for the running system is given in Fig. 2(c). Figure 2(b) shows the rear side of the cart before panel installation. One compartment (item b in the figure) is specifically left empty for keeping the medical tools and system maintenance accessories such as ultrasound gel, distilled water, latex gloves and protective goggles, etc. One optical breadboard (item f in the figure) is bolted on a retractable plate in the cart. The reference arm, DBD, fiber coupler and the polarization controllers for the OCT/OCTA modalities are mounted on the breadboard. Before an imaging session, to optimize the performance of OCT, the operator can pull out the retractable plate for easy access to the breadboard. During imaging, both the operator and the patient wear protective goggles as shown in Fig. 2(c). PAT and OCT/OCTA are performed sequentially with a sensor head switching process in between. Ultrasound gel is used as the acoustic couplant for PAT and index matching material for OCT/OCTA.

**Figure 3:**
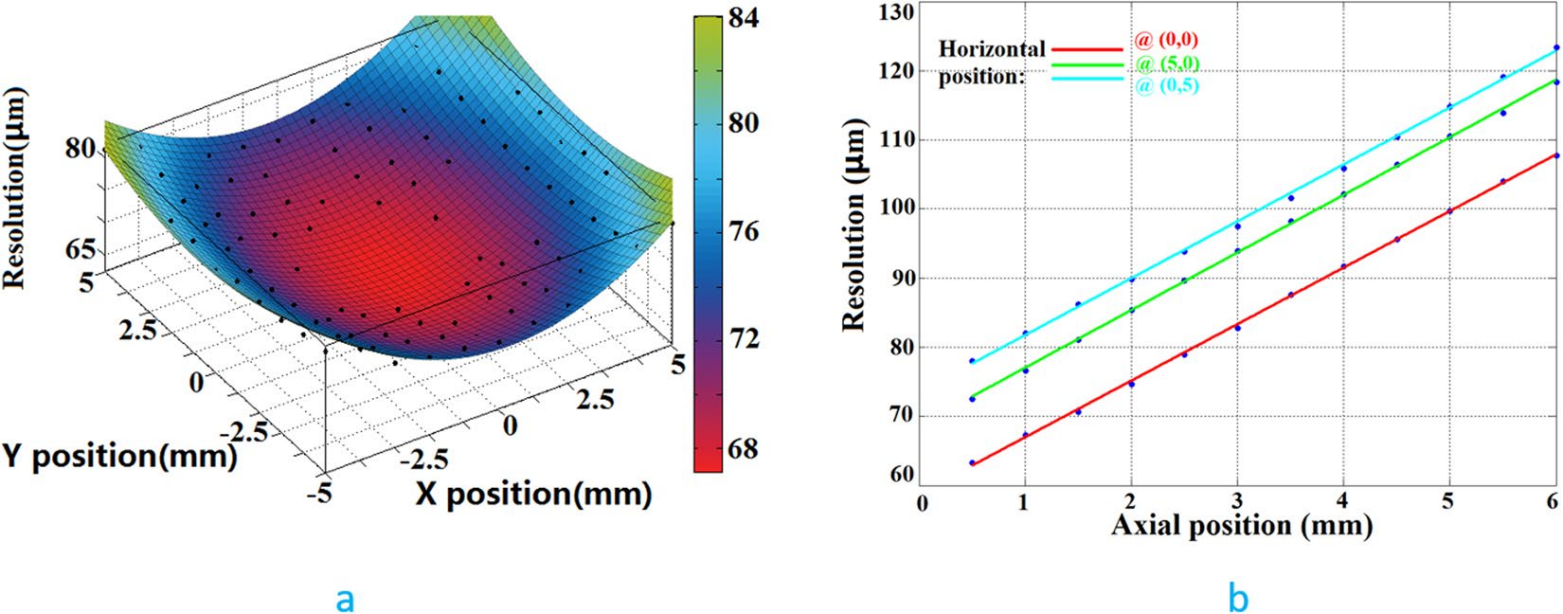
(**a**) Lateral resolution distribution at z = 0.5 mm. (**b**) Lateral resolution change over depth for three (x, y) coordinates.

The control applications for the OCT/OCTA/PAT system are programmed in LabVIEW (2015 & 2016, National Instruments, TX, US). Image reconstruction is performed using MATLAB (R2010a & R2016a, MA, US). The time reversal algorithm provided in the k-Wave toolbox^37^ is used for PAT image reconstruction. Lateral co-registration between the OCT/OCTA volumes and the PAT volume is achieved in ImageJ^38^ by using the transition zone as well as the surface skin pattern^15^. Co-registration in the z direction is achieved in Amira (6.0, FEI, OR, US) by merging the vessels in the transition zone acquired by OCTA and PAT. While the characterization of the OCT/OCTA system is relatively straightforward, the lateral resolution of the PAT system is affected by several parameters. For our cart-based system, we measure the PAT system resolution using the same scan protocols we use for patient imaging – 1 cm × 1 cm range with 70 µm step size. A chrome coated resolution target is placed at different distances to the sensor from 0.5 mm to 6 mm with 0.5 mm increment. One coated square with sharp edges was rotated to align with the x and y axes. For every z step, the same edge of the square is translated in the x and y directions for 5 mm each. Assuming symmetry of spatial resolution over the x and y axes, we use the measured points to cover one quadrant and then mirror the corresponding resolution values to the other three quadrants. Figure 3(a) shows the lateral resolution distribution at 0.5 mm above sensor surface over x-y plane. Figure 3(b) shows how the lateral resolution changes over depth for three representative (x, y) coordinates – (0, 0), red line; (5, 0), green line and (0, 5), blue line. We can see that the best lateral resolution achieved is 62 µm. The axial resolution was measured at (x, y, z) coordinate (0, 0, 0.5) to be 31 µm. A linear curve fitting is used in Fig. 3(b). We noticed that, experimentally, the lateral resolution degenerates almost linearly over the depth range between 0.5 mm to 6 mm, which is comparable to previous characterization results^36^. However, comparing the blue and the green curves, we notice that the lateral resolution is not symmetrically changing in the x and y directions. The lateral resolution in y axis is approximately 5 µm poorer than that in the x axis. This is because the probe design removed the spherical mirrors that were used previously to perform conjugated scanning^29^.

After image reconstruction and volume co-registration, vessel quantification is performed using a skeletonization algorithm-based method^35^. In brief, after pre-processing the volumes with a 3D median filter and a Frangi vesselness filter that can suppress noise and shadow artifacts^39^, the vessels are automatically segmented. The 3D skeleton is then found using a medial axis extraction algorithm^40^. For each of the patient’s data, a region of interest (ROI) covering the diseased zone and an adjacent ROI for the healthy zone are manually selected and the skeleton is searched to extract quantitative vascular parameters. To standardize the vascular pattern quantification, a fixed ROI size that is compatible with the diseased and healthy zones to analyze was chosen. In total, six parameters for the vasculature network can then be extracted in the ROIs, namely the number of vascular trees (NT); the vascular density (VD), which is the ratio of the number of skeleton voxels over the volume of the ROI; the number of branching nodes (NB); 2D distance metric (DM), which is the ratio of the actual path length of a skeletonized vessel over the linear distance between the vessel’s end points, giving an idea of how the vessel path may deviate from straight vessels; ICM, which is the DM multiplied by the number of inflection points found in the vessel’s path; and SOAM, which is a tortuosity parameter that is the sum of all angles of a skeletonized vessel in space. The mathematical description of the tortuosity parameters (DM, ICM, SOAM) can be found in another work^41^. The quantitative vascular analysis is done using the OCTA and the PAT data separately to illustrate vascular features in both the superficial and the deep plexus.

The experiment procedure is approved by the Ethics Committee of the Medical University of Vienna (EK 1246/2013). All experiments followed the guidelines of the protocol approved by this committee. Informed consent is obtained from participating human subjects for the experiment. Only the local diseased regions are photographed without facial area, which cannot lead to identification of the human subjects.

## Results and Discussion

**Figure 4:**
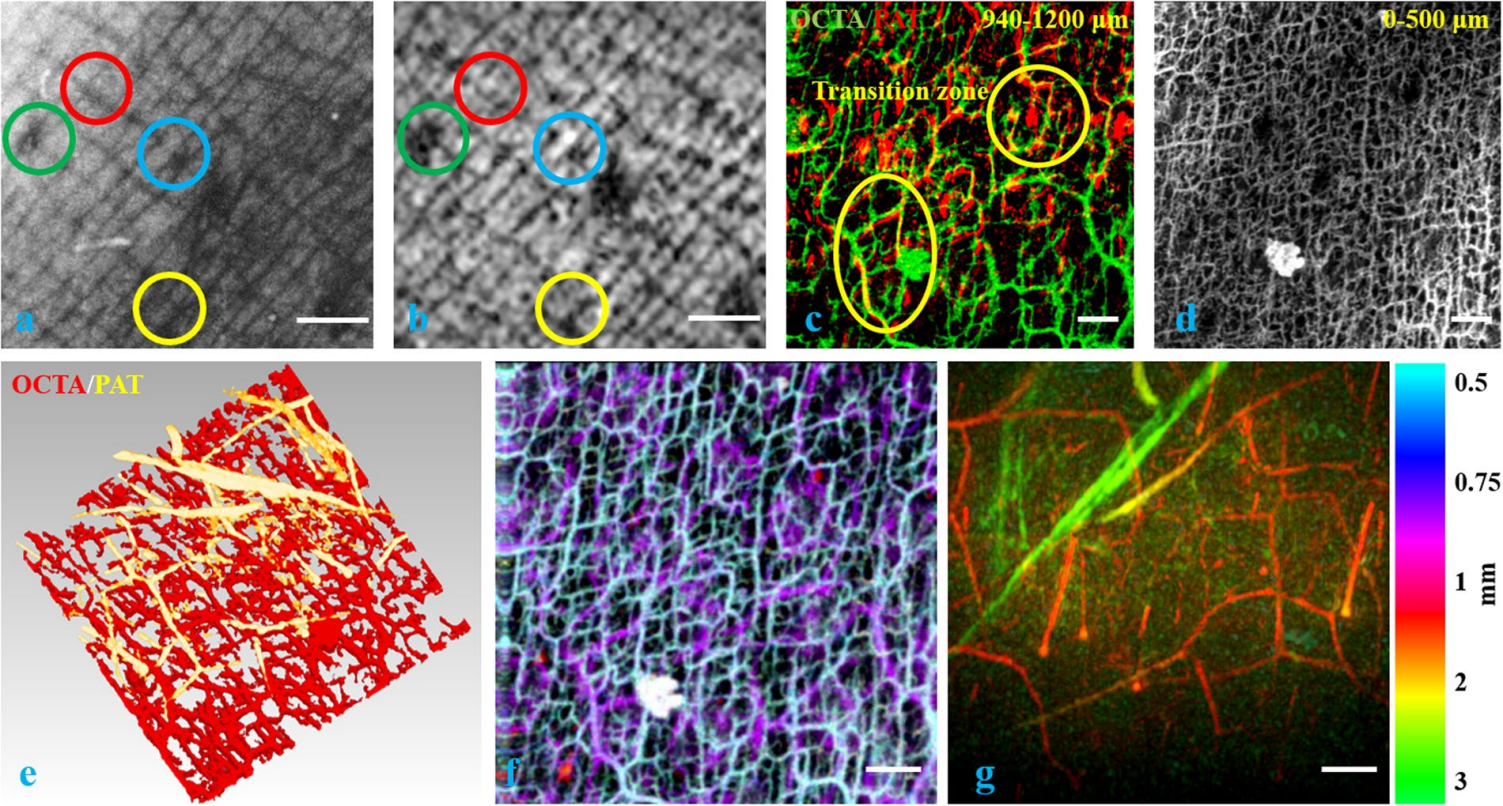
(**a**) Surface topography of the skin given by OCT. (**b**) Absorption from skin surface given by PAT. Four colored circles in (**a**) and (**b**) indicate the same features seen in OCT and PAT. (**c**) Transition zone *en face* view showing the same vessels resolved by both OCTA (green) and PAT (red). (**d**) Skin vasculature in gray scale between the depth range [0 0.5 mm] in z direction imaged by OCTA. (**e**) 3D view in -z direction for the blood vessel network. PAT and OCTA resolved vessels are colored gold and red, respectively. (**f**) Depth color coded image of skin vasculature between [0.5 mm, 1 mm] in z direction using fused OCTA/PAT volume. (**g**) Depth color coded image of skin vasculature between [1 mm, 3 mm] in z direction using fused OCTA/PAT volume. Scale bar = 1 mm.

We imaged a nevus araneus using the combined OCT/OCTA/PAT system. Figure 4(a and b) are *en face* views from OCT and PAT, respectively for the surface of the skin showing the topography. As we can see from the four circle pairs (paired by color), the same features of skin pattern are seen in both modalities. The reason similar patterns are seen in both figures is that the melanin layer in the epidermis follows the topography of skin. Therefore, when a horizontal slice of the PAT volume is extracted from the epidermis region, where melanin is the absorber, we can see similar patterns as are given by *en face* OCT for the skin surface. We use this surface pattern matching as the first level of volume co-registration between OCT/OCTA and PAT. Close to the penetration depth limit of OCTA, we can find a transition zone in which vessels are visualized by both OCTA and PAT. By summing the depth range between 0.94 mm – 1.2 mm into one plane for both OCTA and PAT, we can easily find the common vessels resolved by both modalities. Figure 4(c), in which OCTA is in the green channel and PAT is given in the red channel, shows the common vessels in circled regions. These vessels in this transition zone are found and matched as the second level of volume co-registration, supplementing the skin topography matching, which is used for the first level volume co-registration. Figure 4(d) reveals prolific small blood vessels in the first half millimeter in skin. Figure 4(e) is a volumetric view from the -z perspective showing the complete vasculature network. Figure 4(f and g) are blood vessels in the depth range 0.5 mm – 1 mm and 1 mm – 3 mm in the z direction, respectively. These two images are color coded in depth with the hue-depth projection bar given to the right of Fig. 4(g).

**Figure 5:**
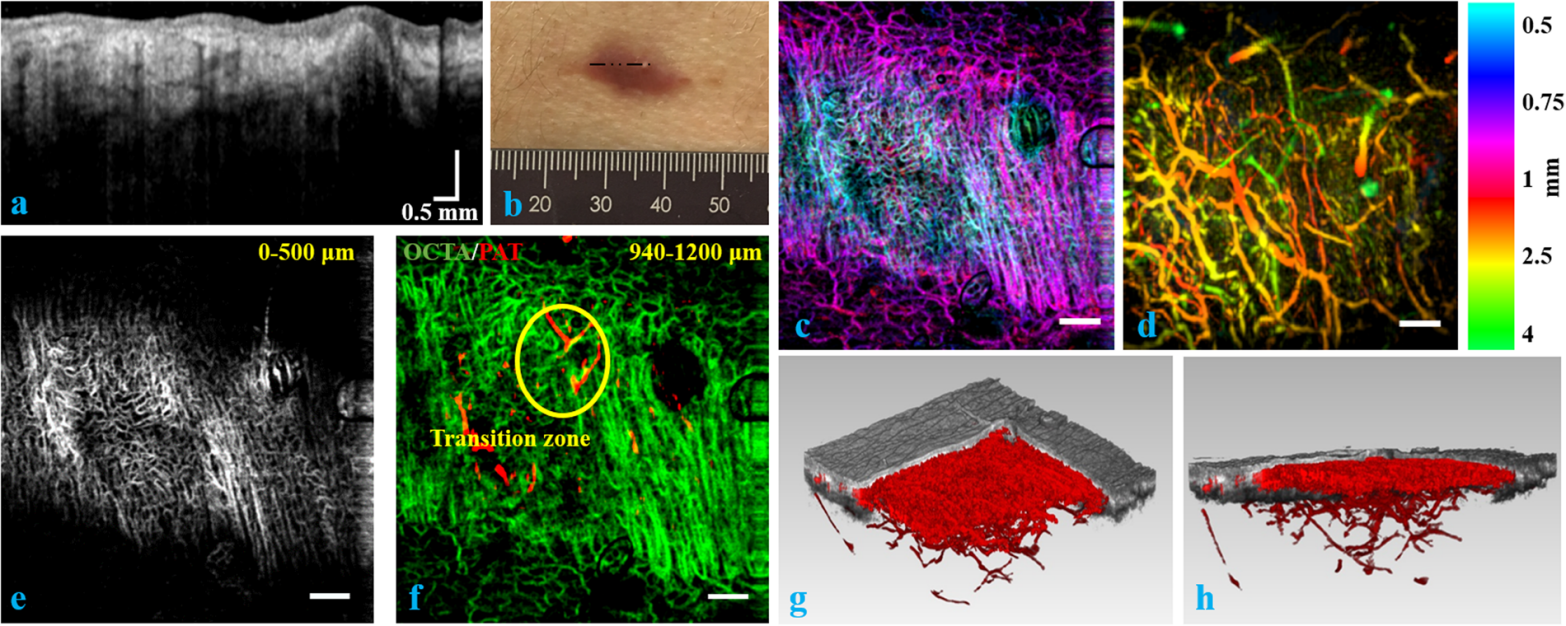
(**a**) OCT B scan of the skin over the dashed line in (**b**). (**b**) Photo of the surgical scar. (**c**) Depth color coded image of skin vasculature between [0.5 mm, 1 mm] in z direction using fused OCTA/PAT volume. (**d**) Depth color coded image of skin vasculature between [1 mm, 4 mm] in z direction using fused OCTA/PAT volume. (**e**) Skin vasculature in gray scale between [0 0.5 mm] in z direction imaged by OCTA. (**f**) Transition zone *en face* view showing the same vessels resolved by both OCTA (green) and PAT (red). (**g**) and (**h**) OCT/OCTA/PAT merged volume 3D rendering at two perspectives. Scale bar = 1 mm except in (**a**).

We imaged another patient one month after his surgery on the lower back to assess wound healing. The results are given in Fig. 5. Figure 5(a) shows an OCT B scan over the dashed line in Fig. 5(b), which is a photo of the scar at the site of surgery. Figure 5(c and d) are depth color coded images showing the blood vessels in the depth ranges of 0.5 mm – 1 mm and 1 mm – 4 mm, respectively. In Fig. 5(e), we see dense microvasculature in the operated area. Similar as Figs 4(c) and 5(f) shows the transition zone of blood vessels from OCTA to PAT. Notwithstanding the limited effective penetration depth of OCT demonstrated in Fig. 5(a), we can still visualize many vessels using OCTA deeper than 0.94 mm in Fig. 5(f). We assume that this is partially caused by shadow artifacts in OCTA, which make shallower vessels reconstructed in deeper zones, and partially because the OCTA reconstruction algorithm is sensitive enough to resolve deeper vessels where morphological OCT already reaches its penetration depth limit. In additional to the tissue morphology seen in OCT, we can distinguish the vascular pattern in the scar from its surrounding area as seen in Fig. 5(c). Figure 5(g and h) display the OCT/OCTA/PAT merged volume in 3D at two different perspectives with OCT shown in gray. These two sub-figures also demonstrate the location of the superficial plexus in the skin and how the combined system visualizes deeper vessels.

**Figure 6:**
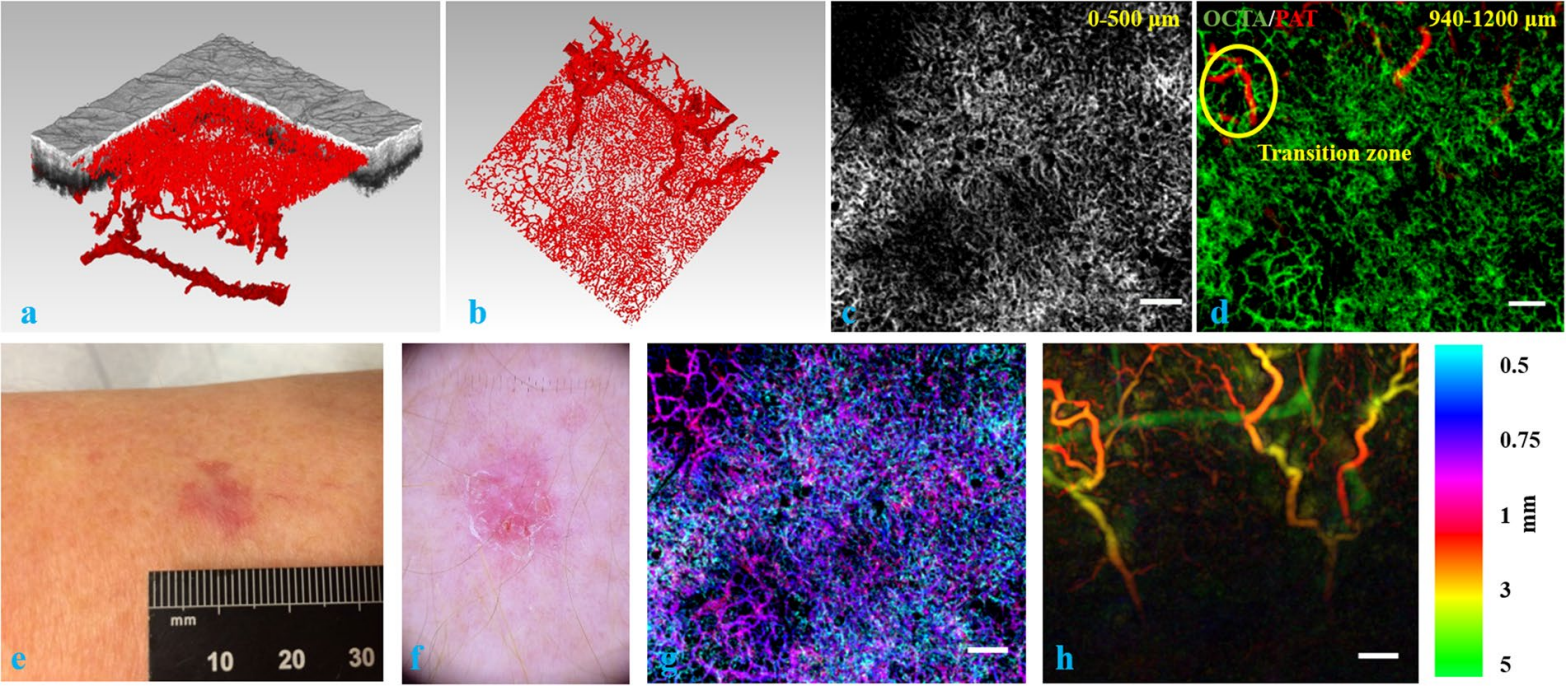
(**a**) Volumetric rendering of the fused OCT/OCTA/PAT volume. (**b**) Top view of the vasculature network. (**c**) Skin vasculature in gray scale between the depth range [0 0.5 mm] in z direction imaged by OCTA. (**d**) Transition zone between [0.94 mm, 1.2 mm] in z direction with OCTA in green and PAT in red. (**e**) Clinical image of a shiny pink macule on the thigh of a patient. (**f**) Dermatoscopy image of the same lesion. (**g**) Depth color coded image of skin vasculature between [0.5 mm, 1 mm] in z direction using fused OCTA/PAT volume. (**h**) Depth color coded image of skin vasculature between [1 mm, 5 mm] in z direction using fused OCTA/PAT volume. Scale bar = 1 mm.

Finally, we imaged a superficial basal cell carcinoma on the thigh of a patient. Figure 6(a) shows a general view of the OCT/OCTA/PAT fused volume, in which we can clearly see the plexus of blood vessels in skin – the superficial plexus and deep plexus that run relatively parallel to the x-y plane, and the connecting vessels in which blood vessels branching off from the deep plexus to feed the superficial plexus. Figure 6(b) shows a top view of the vasculature network. Figure 6(c) gives the vasculature in the first 500 µm in skin revealed by OCTA. Figure 6(d) again demonstrates the transition zone from OCTA to PAT in the z direction. Figure 6(e) presents the clinical image of the lesion on the thigh of the patient. Figure 6(f) shows the dermatoscopy image of the lesion. It reveals a white to pink structureless area with erosions and thin serpentine vessels. Figure 6(g and h) are depth color coded images for the depth ranges of 0.5 mm – 1 mm and 1 mm – 5 mm in z direction measured from the surface of the skin, respectively.

**Figure 7:**
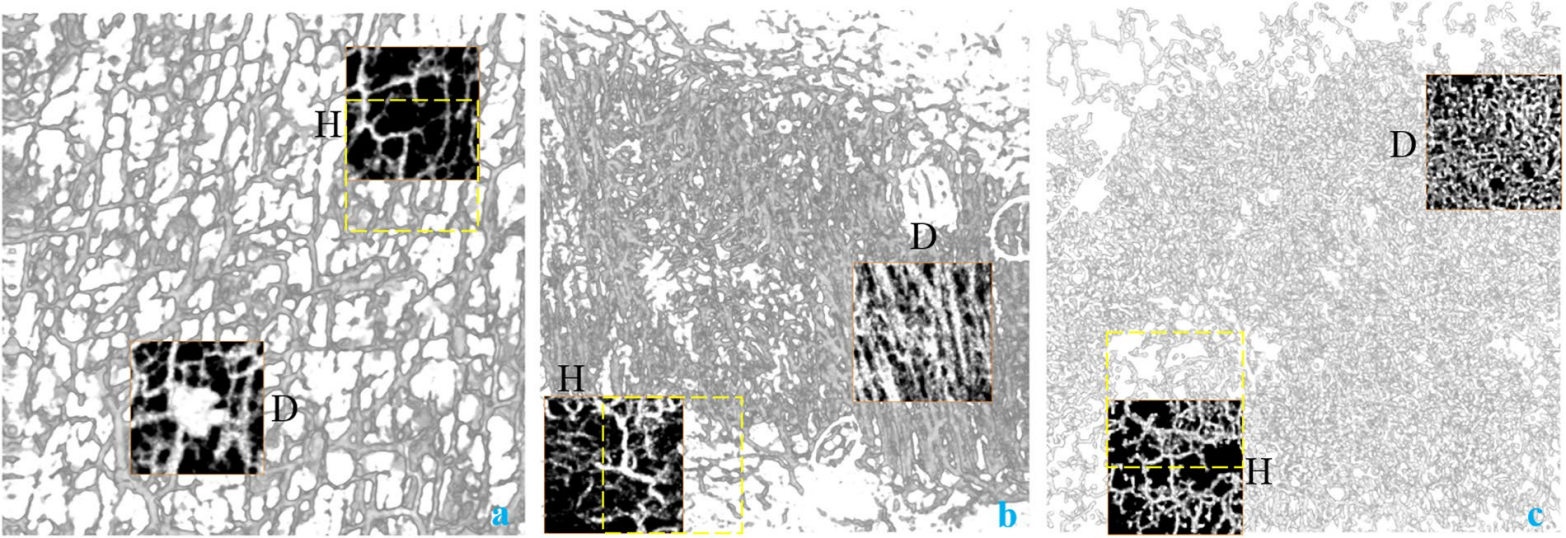
The ROIs chosen for comparison between the healthy and diseased zones for nevus araneus (**a**), scar tissue (**b**), and basal cell carcinoma (**c**). Yellow dashed squares are the second healthy ROIs used in Table. 3.

Quantification results for the three patients’ vasculature networks from the capillary loops to the vessels in the transition zones (i.e. using the images acquired with OCTA) are given in Table 1. Table 2, on the other hand, shows the results from the vasculature networks from the transition zone to the final depth. The regions we use for comparing the diseased and healthy zones are indicated below in Fig. 7. The grayed-out vessels in Fig. 7 are from OCTA. The zones with black background are the ROIs chosen for comparison between the healthy and diseased zones (marked with “H” for healthy and “D” for diseased on the side of the ROIs). All the ROIs are manually selected based on the vascular pattern resolved in OCTA.

**Table 1:** Quantification of the Superficial Vasculature in the Three Patients (OCTA).

	Healthy Area/Diseased Area					
	NT	VD	NB	DM	ICM	SOAM
NA	4/1	0.0015/0.0039	54/126	2.2781/3.4381	40.923/93.761	0.1848/0.1152
SS	8/4	0.0018/0.0035	108/232	1.9082/2.4158	34.625/48.748	0.1683/0.2496
BCC	4/1	0.0045/0.0071	280/273	2.8176/2.99	64.339/117.39	0.1989/0.2021

**Table 2:** Quantification of the Deep Vasculature in the Three Patients (PAT).

	Healthy Area/Diseased Area					
	NT	VD	NB	DM	ICM	SOAM
NA	5/1	0.0004/0.0005	15/19	1.2988/1.6925	15.218/48.854	0.0224/0.096
SS	4/3	0.0004/0.0008	11/35	2.009/2.4102	29.204/52.402	0.0674/0.0591
BCC	5/3	0.0008/0.0015	35/59	2.1974/2.2106	26.102/54.288	0.0612/0.0637

**Table 3:** Quantification Using a Second Healthy Area (OCTA).

	Healthy Area/Diseased Area					
	NT	VD	NB	DM	ICM	SOAM
NA	3/1	0.0022/0.0039	82/126	2.7059/3.4381	63.066/93.761	0.16/0.1152
SS	3/4	0.0026/0.0035	93/232	2.5042/2.4158	60.645/48.748	0.292/0.2496
BCC	1/1	0.0055/0.0071	209/273	2.9705/2.99	81.923/117.39	0.251/0.2021

In Tables 1 and 2, all six parameters are given for the six volumes (3 for OCTA, 3 for PAT) in both the healthy area and the diseased area. The acronyms are NA: nevus araneus, SS: surgical scar, BCC: basal cell carcinoma, NT: number of vascular trees, VD: vascular density, ICM: inflection count metric, SOAM: sum of angles metric. A comprehensive statistical analysis is not yet possible due to the limited number of cases analyzed.

Even though we only applied the system in a small number of cases, we demonstrate some significant differences between healthy and diseased areas. Firstly, we can see that the NT is typically less in the diseased area, but the NB and the VD are typically higher in these areas. This means that the diseased area normally presents a larger total number of vessels, with an intricate architecture and more branching points. The tortuosity parameters also confirm the vascular complexity in the diseased areas. Noticeably, we can see that the ICM is normally higher in diseased areas, which shows that, when compared to healthy vasculature, the blood vessels in the diseased area have more twists and turns as well as a higher chance for deviation from orderly vascular network. The SOAM, instead, does not have a contingent indicating value in the three cases. As a metric that highlights the high frequency, low amplitude tight coil vessels typical in tumor lesion vasculature, SOAM’s value in our application requires more cases of skin cancer to be investigated.

A variability check for the six parameters is also done for the OCTA data by choosing another healthy area next to the ones indicated by “H” in Fig. 7 with partial overlap for each case. This second healthy area is delineated by the dashed line in Fig. 7. Table 3 gives the results acquired using this second healthy area. The mismatch in NT between Tables 1 and 3 is attributed to the fact that different areas cut the blood vessels in different points. We can see that all the six parameters for NA in the newly selected area still have similar differences compared to the diseased areas. For SS, NB is changed slightly but DM, ICM and SOAM become greater in the healthy area than in the diseased area. In the BCC case, VD, DM and ICM stayed in the same trend for the newly selected area while NB and SOAM see a relatively big dependence on ROI selection. As a first trial of vessel quantification using OCTA/PAT, there is subjectivity when choosing ROIs. With more patients studied, we believe the subjectivity can be minimized when we image the same type of disease and perform piecewise quantification in the full scanning range. From the variability check using OCTA data, we notice that for different diseases the vital quantification parameters vary. This also requires us to further explore the quantification parameter – disease specification relationship in future studies.

Currently, we rely on labor intensive visual inspection to do the volume co-registration work. This is time consuming and increases the turnover time of the system in clinical application. For regions with well-defined skin topography, such as the case shown in Fig. 4(a and b), an automatic pattern matching algorithm might assist volume co-registration. For anatomic sites or skin conditions that do not bear surface topography similarities between the OCT and PAT volumes, we need to explore possibilities of automatic vessel co-registration in the transition zone. In Figs 5(f) and 6(d), we notice that the transition zone may only bear very limited overlap between OCTA and PAT. This is because a few vertical feeding vessels branching off the dermal plexus can already support a relatively large network of superficial plexus vessels, which is demonstrated explicitly in the 3D renderings such as Fig. 6(b).

From Fig. 6(a), we notice that the imaging area was not sufficient to see the specific connecting reticular dermal vessels between the deepest resolved vessel and the superficial plexus. This will induce inaccuracy in the extraction of quantitative parameters from the vasculature structure for the PAT volume, which may result in unreliable results for the vascular quantitative analysis. But due to the limited repetition rate of the excitation laser sources, larger imaging area requires more time, which negatively affects the image quality due to motion artifacts. Empirically, imaging extremities for several minutes does not pose a significant challenge. As is shown in Fig. 2(c), a vacuum splint in most cases is sufficient to support and immobilize limbs. In our imaging sessions, hand, arm, leg and foot can be imaged without much motion blur. In the neck and the trunk of the body, however, aspiration and heartbeat require us to reduce the imaging time for a higher success rate. Currently we are investigating compressed sensing methods^42^ as well as parallel detection possibilities to reduce the imaging time by one order of magnitude. The pressure exerted on skin is another parameter that needs to be more accurately controlled. During our experiments we noticed that the pressure level affects the final image quality. We assume that too high pressure hinders blood flow while too low pressure will not compress the skin sufficiently to maximize the effective imaging depth. In the next generation probe design, we plan to incorporate a miniaturized force measuring device juxtaposed to the polymer film sensor so that we can experimentally determine the optimal pressure on skin for PAT imaging.

As the first ever bedside non-invasive human cutaneous vasculature imaging system, the prototype still has room for improvements. In addition to the vacuum splint, we are also designing a new probe with 3D precision printing that can be light weight and therefore allows easier adjustment. The makeshift articulated arm will also be replaced with a floor-mounted robotic arm for robust and accurate positioning. The ability to perform a spectroscopic analysis of multiwavelength photoacoustic images is also being explored by exploiting the per pulse wavelength tuning feature of the excitation laser.

## Conclusions

We have assembled a mobile cart-based OCT/OCTA/PAT system for clinical translational studies. Compared to the previous version multimodal system, the new cart-based system enables bedside imaging in a clinical ward, greatly shortens patient throughput time, therefore permits easier enrollment of human subjects. Without the need of a fixed pressure exchanging water cooling system, the new excitation source for PAT, which does not require constant nitrogen purge and flash lamp monitoring, significantly alleviates the maintenance costs of the system. By merging the DAQs into one workstation and sharing some electronic devices for the sub-systems, the operation of the multimodal system has been simplified, meaning that clinicians can be trained to perform imaging without much technical assistance. The multimodal imaging system has been applied to patients with nevus araneus, a surgical scar and basal cell carcinoma. The cutaneous vasculature extracted after the imaging session was then quantified using a skelotonization algorithm-based method. The results demonstrate that our system can non-invasively extract the complete human blood vessel network in skin with various morphological or vascular malformations. Thanks to the analysis of the vascular complexity, a quantitative differentiation between healthy and diseased tissues is possible when considering both the morphological and tortuosity parameters for the blood vessel networks. In the future, by adding a spectroscopic capability to PAT, we expect to have a mobile non-invasive functional multimodal *in vivo* imaging platform for human skin, giving the specialists the information of tissue morphology, complete vasculature network, vessel feature quantification, blood oxygenation level, etc. at the same time. With an anticipated higher throughput of patients using the cart-based system, we believe that the OCT/OCTA/PAT system will be a valuable tool for clinical diagnosis and prognosis.
