# Author Correction: Non-invasive multimodal optical coherence and photoacoustic tomography for human skin imaging

**DOI:** 10.1038/s41598-018-31225-8

**Published:** 2018-08-30

**Authors:** Zhe Chen, Elisabet Rank, Kristen M. Meiburger, Christoph Sinz, Andreas Hodul, Edward Zhang, Erich Hoover, Micheal Minneman, Jason Ensher, Paul C. Beard, Harald Kittler, Rainer A. Leitgeb, Wolfgang Drexler, Mengyang Liu

**Affiliations:** 1 Center for Medical Physics and Biomedical Engineering, Medical University of Vienna, Währinger Gürtel 18-20, AKH 4L, 1090 Vienna, Austria; 2 Dipartimento di Elettronica e Telecomunicazioni, Biolab, Politecnico di Torino, Corso Duca degli Abruzzi 24, 10129 Torino, Italy; 3 Department of Dermatology, Medical University of Vienna, Währinger Gürtel 18-20, AKH 7J, 1090 Vienna, Austria; 4 Department of Medical Physics and Biomedical Engineering, University College London, Gower Street, WC1E 6BT London, UK; 5 Insight Photonic Solutions, Inc., 2650 Crescent Drive, Number 201, Lafayette, CO 80026 USA

Correction to: *Scientific Reports* 10.1038/s41598-017-18331-9, published online 21 December 2017

This Article contains typographical errors in the Acknowledgements section.

“Horizon 2020 Framework Programme (H2020) (792720)”

should read:

“Horizon 2020 Framework Programme (H2020) (732720)”

